# Transient perivascular inflammation of the carotid artery superimposed on carotid atherosclerotic plaque, recurring at the mid-common carotid artery: a case report

**DOI:** 10.3389/fcvm.2026.1913022

**Published:** 2026-10-29

**Authors:** Hongru Feng, Lili Yu, Wenyi Li, Xiaoliang Wang

**Affiliations:** 1 Department of Ultrasound, The Second Hospital of Hebei Medical University, Shijiazhuang, China; 2 Department of Neurology, The Second Hospital of Hebei Medical University, Shijiazhuang, China; 3 Department of Neurosurgery, The Second Hospital of Hebei Medical University, Shijiazhuang, China

**Keywords:** carotid plaque, neck pain, perivascular infiltration, transient perivascular inflammation of the carotid artery, ultrasonography

## Abstract

Transient perivascular inflammation of the carotid artery (TIPIC) syndrome is a rare acute cervical pain syndrome of unclear etiology and pathogenesis. Its main symptom is unilateral neck pain, typically localized to the distal common carotid artery bifurcation, and symptoms usually last for less than two weeks. The syndrome is self-limiting and non-steroidal anti-inflammatory drugs (NSAIDs) are the mainstay of treatment. We report a case of a 37-year-old woman presenting with persistent bilateral neck pain radiating to the parotid region, in whom imaging confirmed a unilateral lesion of the left common carotid artery (bilateral symptoms with unilateral TIPIC). The patient experienced two separate episodes of acute pain at the same site (mid-portion of the left common carotid artery) within six months, in the setting of a pre-existing plaque at that location. Imaging examinations, including ultrasonography, plain magnetic resonance imaging (MRI), and high-resolution vessel wall MRI, clearly demonstrated perivascular inflammation at the mid-portion of the left common carotid artery, rather than thickening of the underlying plaque. Following a 2-week course of NSAID treatment, resolution of the perivascular lesion was observed after both episodes. Based on the clinical features, imaging findings, and established diagnostic criteria, a diagnosis of TIPIC syndrome was confirmed. Because of its transient clinical course and moderate pain intensity, this is often misdiagnosed, even by neurologists. This case is distinguished by two features: the lesion occurred in the mid-portion of the common carotid artery rather than the more common distal bifurcation, and it was superimposed on a pre-existing carotid atherosclerotic plaque. The atypical localization and plaque association broaden the recognized phenotypic spectrum of this rare condition.

## Introduction

Transient perivascular inflammation of the carotid artery (TIPIC) syndrome is a rare, unclassified vascular disorder. It typically affects the distal bifurcation of the common carotid artery, and its clinical manifestation is usually pain in the lateral neck and carotid bifurcation area, predominantly unilateral. Lecler et al. (1) first proposed the more appropriate term “TIPIC syndrome” based on clinico-radiological features and established precise diagnostic criteria: 1) Acute pain on the carotid surface, with or without radiation to the head; 2) Imaging showing eccentric perivascular infiltration; 3) Imaging excluding other (non-)vascular diseases; 4) Improvement within 14 days, either spontaneously or after anti-inflammatory treatment. A secondary diagnostic criterion is spontaneous resolution of the inflammatory lesion.

## Case report

In April 2025, a 37-year-old woman presented to the Neurosurgery Department of the Second Hospital of Hebei Medical University, with persistent bilateral neck pain radiating to the parotid region for 2 days, exacerbated by neck extension and palpation over the mid-portion of the left common carotid artery. Her medical history included hyperlipidemia and a known plaque at the mid-portion of the left common carotid artery. She denied recent infection, trauma, surgery, epidemic disease, and had no history of migraine or other neurological symptoms. Physical examination revealed no neck swelling, asymmetry, or bruising, Localized tenderness was noted upon palpation over the mid-portion of the left common carotid artery, along with hyperalgesia in bilateral neck-to-parotid regions and paresthesia of the left ear. The remaining ear, nose, and throat (ENT) examination was unremarkable, and no crepitus or pain was elicited on temporomandibular joint palpation. The rest of the physical and neurological examinations were unremarkable.

Ultrasonography showed localized, eccentric, hypoechoic thickening involving both the medial wall/plaque and the adjacent periadventitial fat plane at the mid-portion of the left common carotid artery (corresponding to the tender point), with poorly defined borders, measuring approximately 4.3 mm in maximal thickness (Figures 1A,B) and 32.8 mm in length, causing mild luminal stenosis, with no evidence of dissection. Because low-echoic plaque and plaque-surrounding inflammation both appear as dark/anechoic areas on conventional B-mode ultrasound, the two components could not be fully separated; the periadventitial nature of the inflammatory component was corroborated by contrast-enhanced high-resolution vessel-wall MRI (Figure 1F). Ultrasound examinations were performed on Philips iU Elite and GE LOGIQ E8 systems, both equipped with high-frequency linear-array and convex-array probes. All ultrasound measurements were independently obtained by two attending physicians, each with more than 10 years of experience in vascular ultrasound, who were blinded to each other's results.

**Figure 1:**
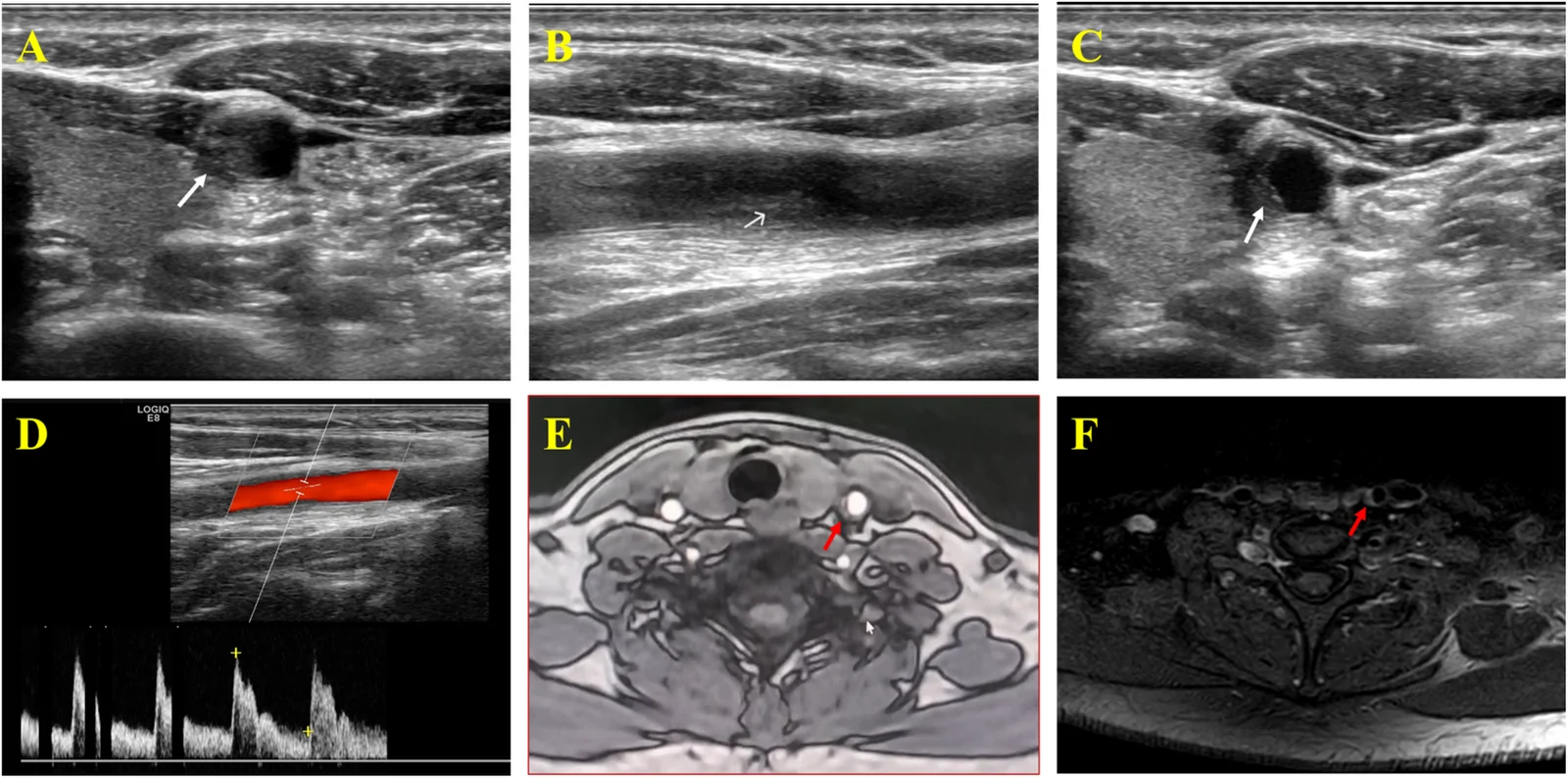
**(A, B)** (ultrasound): localized thickening of the medial wall of the mid-portion of the left common carotid artery, measuring up to 4.3 mm thick and approximately 32.8 mm long, with an unclear border to the normal wall. **(C)** (One-month follow-up ultrasound): Significant reduction in the wall thickening, with the thickest part measuring about 2.5 mm. **(D)** (Ultrasound): Normal blood flow spectrum at the site of localized wall thickening in the mid-portion of the left common carotid artery. **(E)** (Plain MRI): Isointense signal on T1-weighted images and slightly hyperintense signal on T2-weighted images in the wall of the mid-portion of the left common carotid artery. **(F)** (High-resolution MRI): Eccentric enhancement around the wall of the mid-portion of the left common carotid artery, representing perivascular infiltrative changes with unclear borders.

These findings were consistent with perivascular inflammation. Color Doppler flow imaging (CDFI) showed altered flow signal at the site of thickening, corresponding to mild luminal narrowing caused by the eccentric wall and perivascular thickening protruding into the lumen rather than an intraluminal or mural defect; no dissection flap, thrombus, or flow-altering lesion was seen. Pulsed-wave Doppler (PWD) revealed a normal carotid artery flow spectrum: peak systolic velocity (PSV) 93.4 cm/s, end-diastolic velocity (EDV) 25.8 cm/s, resistance index (RI) 0.72 (Figure 1C). Examination of the right side of the neck was normal. Additional ultrasound showed no significant abnormalities in the parotid glands, submandibular glands, cervical/or supraclavicular lymph nodes, or thyroid. Plain MRI showed an isointense signal on T1-weighted images and a slightly hyperintense signal on T2-weighted images in the wall of the mid-portion of the left common carotid artery (Figure 1D). High-resolution 3 T vessel wall MRI revealed eccentric fusiform thickening of the vessel wall at that site. Contrast-enhanced T1-weighted MRI showed eccentric enhancement around the arterial wall, representing perivascular infiltrative changes with poorly defined borders (Figure 1E). Laboratory tests showed a slightly elevated absolute basophil count with a normal percentage. Urinalysis, liver/and kidney function, blood glucose, glycated hemoglobin, homocysteine, thyroid function, erythrocyte sedimentation rate, anti-streptolysin O, C-reactive protein, and rheumatoid factor were all within normal limits. After differential diagnosis excluded carotid atherosclerotic plaque, subacute thyroiditis, carotid artery dissection, and Takayasu arteritis, TIPIC syndrome was highly suspected. The patient received ibuprofen 300 mg orally [three times daily] for 2 weeks (authors to confirm the exact dose and schedule), after which her symptoms completely resolved. A one-month follow-up ultrasound showed significant reduction in the wall thickening to approximately 2.5 mm (Figure 1F). Six months after the initial episode, the patient experienced similar left neck pain again, with no new trauma or other triggers. MRI was not repeated during this recurrence because the patient declined further cross-sectional imaging; the diagnosis of the recurrent lesion was therefore based on (i) recurrence of identical clinical symptoms at the same anatomical site, (ii) ultrasound demonstrating a hypoechoic, eccentric lesion at that site measuring approximately 3.1 mm in thickness (Figure 2A), (iii) exclusion of carotid dissection and other mimics on clinical and sonographic grounds, and (iv) complete resolution of symptoms after a 2-week course of NSAIDs, with a one-year follow-up ultrasound showing return to the pre-morbid state, with only the original carotid plaque visible. No laboratory tests, including inflammatory markers, were repeated during this episode. Another 2-week course of NSAIDs led to complete symptom resolution. A one-year follow-up ultrasound showed a return to the pre-morbid state, with only the original carotid plaque visible (Figures 2B,C). An ultrasound image obtained four months before the first episode is provided for comparison (Figure 2D). Based on the clinical presentation, imaging features, and diagnostic criteria, the diagnosis of TIPIC syndrome was confirmed.

**Figure 2:**
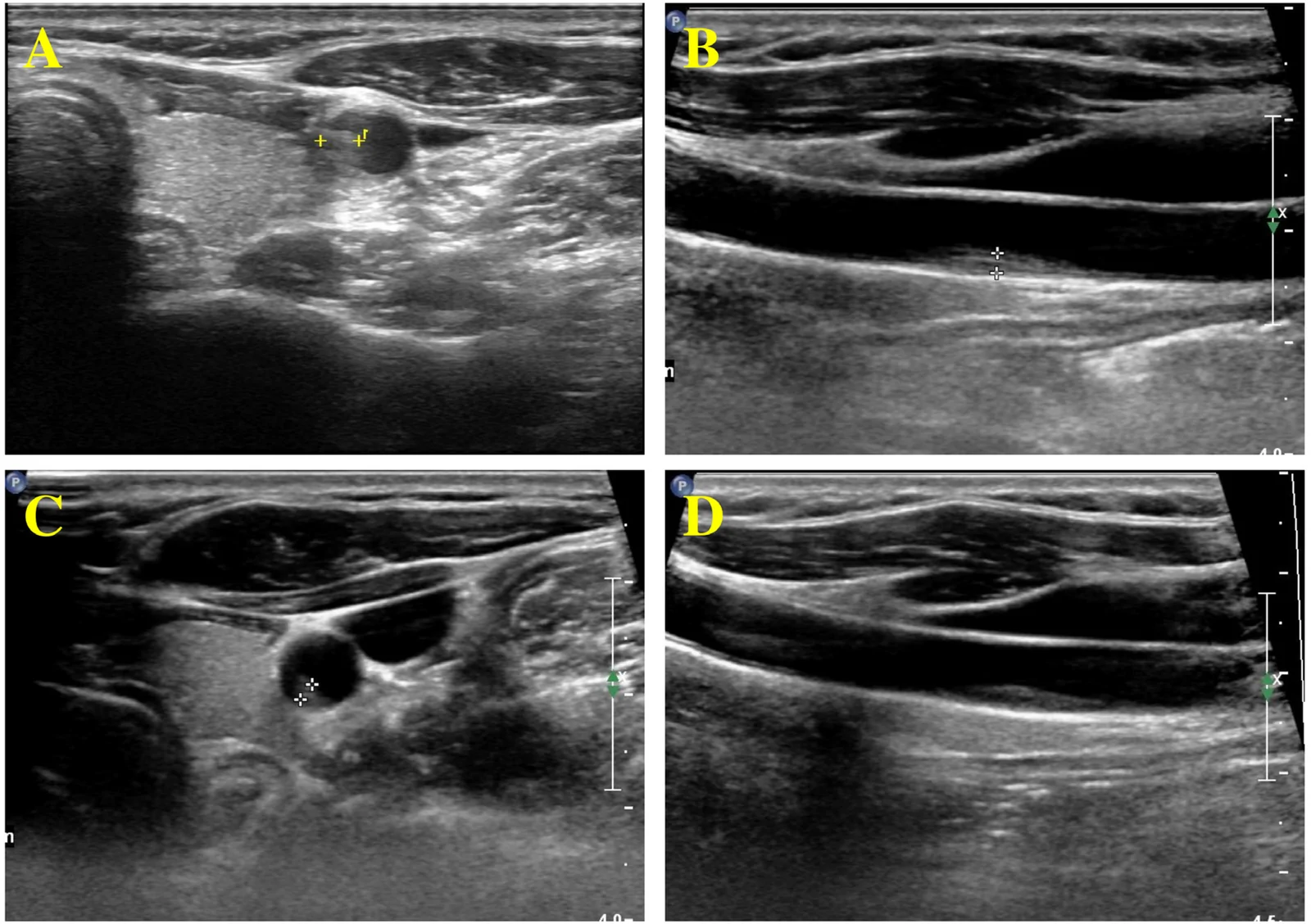
**(A)** (recurrence): localized thickening of the medial wall of the mid-portion of the left common carotid artery, measuring up to 3.1 mm thick. **(B, C)** (One-year follow-up ultrasound, longitudinal and transverse views): The medial wall of the mid-portion of the left common carotid artery appears normal, with only the pre-existing carotid plaque visible. **(D)** (Ultrasound from 4 months before onset): Showing the pre-existing carotid plaque.

As summarized in Table 1, the entire clinical course spanned approximately one year, with two symptomatic episodes at the same mid-CCA site​ and a pre-existing atherosclerotic plaque evident on ultrasound four months before the first episode. Serial ultrasound at matched display settings documented regression of the acute wall thickening to 2.5 mm at one month and normalization at one year, while the pre-existing plaque remained unchanged. It should be acknowledged that, because residual thickening (approximately 2.5 mm) was present at the one-month follow-up, complete resolution before the second episode could not be demonstrated. The change from 2.5 to 3.1 mm may therefore represent true recurrence, persistent inflammation, or measurement variability.

**Table 1:** Timeline of the clinical course and imaging findings.

Time point	Clinical event/imaging finding
∼4 months before 1st episode	Baseline ultrasound: pre-existing carotid plaque only (Figure 2D).
April 2025 (initial episode)	Bilateral neck pain radiating to the parotid region; ultrasound 4.3 mm; plain + high-resolution MRI; 2-week course of ibuprofen.
1 month after 1st episode	Follow-up ultrasound: residual wall thickening ∼2.5 mm (Figure 1C).
∼6 months after 1st episode	Recurrent left neck pain; ultrasound ∼3.1 mm at the same site (Figure 2A); NSAIDs; no MRI.
1 year after 1st episode	Ultrasound: return to the pre-morbid state, with only the original plaque visible (Figure 2B,C).

## Discussion

TIPIC syndrome is a rare condition, with a single-center prevalence of 2.8% among patients with acute neck pain (1, 2). The recurrence rate is reported to be 19% (1); thus recurrence *per se* is not novel. What is distinctive in the present case is the site of recurrence (mid-portion of the common carotid artery rather than the typical distal bifurcation) and its superimposition on a pre-existing atherosclerotic plaque. The median patient age is 48 years (range 39–56 years), with a slight female predominance (female-to-male ratio, approximately 1.5:1) (1). The etiology and pathogenesis remain unclear but may be related to inflammation of unknown origin, certain medications, variants of large-vessel vasculitis, or autoimmune responses (3, 4).Clinically, it typically presents as pain in the lateral neck and carotid bifurcation area, mostly unilateral, occasionally radiating to the ipsilateral ear. Bilateral pain occurs in 8% of cases (5). The present case adds to this minority phenotype: the patient had bilateral symptoms with unilateral TIPIC; no right-sided lesion was demonstrated. The pain is often dull or throbbing, possibly related to edema or distension of the affected area. Less than 20% of patients may have neurological symptoms such as dizziness, vertical diplopia with oculomotor palsy, or sensory/ or motor deficits (6–8). Other possible symptoms include lymphadenopathy (17%), local edema (29%), headache (11%), and fever (6%) (5). Pain worsens with palpation or head/ and neck movement. The course is usually self-limiting, with a good response to NSAID treatment (9).

The syndrome was first described by Dr. Fay in 1927, characterized by tenderness and pain at the carotid bifurcation (10), and was termed “carotidynia.” It was listed as an atypical headache syndrome in the 1988 International Classification of Headache Disorders (ICHD) and classified as an idiopathic syndrome associated with neck pain in the first edition of the ICHD (1). In 2017, Lecler et al. proposed the term “TIPIC syndrome” and established the precise diagnostic criteria mentioned above.

The pathology of TIPIC syndrome involves not only the perivascular tissue but also the arterial wall itself. On grayscale ultrasound, it typically appears as eccentric, hypoechoic thickening around the carotid bifurcation wall, extending into the internal or external carotid artery (5), often with an indistinct wall structure, sometimes showing an “onion-skin” pattern (7). Studies have shown a positive correlation between lesion length and thickness (2). The lumen is usually not stenotic or only mildly stenotic (1), and carotid hemodynamics are typically normal (5). In this case, the clinical presentation was bilateral neck-to-parotid pain, exacerbated by neck extension and palpation over the mid-portion of the left common carotid artery, with a tender point at that location. Ultrasound showed eccentric, hypoechoic thickening at the medial wall of the mid-portion of the left common carotid artery (the original plaque site) with poorly defined borders, causing luminal stenosis but no significant hemodynamic changes on color Doppler. Plain MRI showed isointense T1-weighted and slightly hyperintense T2-weighted signals. High-resolution 3 T vessel wall MRI revealed eccentric fusiform thickening, and contrast-enhanced T1-weighted MRI showed eccentric perivascular enhancement. The patient's symptoms resolved after two weeks of ibuprofen treatment during both episodes, with follow-up ultrasound showing a return to the pre-morbid state. The diagnosis was clear based on the typical clinical and imaging presentation, improvement after anti-inflammatory treatment, and resolution on one-year follow-up.

A mechanistic question raised by this case is why TIPIC occurred at the site of a pre-existing atherosclerotic plaque in the mid-portion of the common carotid artery rather than at the typical distal carotid bifurcation. Atherosclerotic plaques are commonly accompanied by neovascularization of the vasa vasorum and structural abnormalities of these microvessels, whereas TIPIC is characterized by inflammation targeting the adventitia and periadventitial tissue (11, 12). We therefore speculate that, in a patient with systemic immune triggers for TIPIC, the abnormally proliferated vasa vasorum network at the plaque site may serve as a conduit for inflammatory-cell infiltration, thereby acting as a “weak point” that influences the localization of inflammation. This same framework may also bear on recurrence, if the persistent altered microvascular milieu at the plaque site renders it susceptible to repeat inflammatory episodes. It must be emphasized that no direct evidence currently links atherosclerotic plaque or vasa vasorum changes to the pathogenesis or localization of TIPIC; this proposed association remains speculative and requires further investigation.

The diagnosis of TIPIC syndrome requires exclusion of the following differential diagnoses: (1) Carotid atherosclerotic plaque: On ultrasound, plaques are located in the intima (13), often calcified (14), and can cause stenosis and hemodynamic changes. Although this patient had hyperlipidemia and a known plaque at the mid-portion of the left common carotid artery, the lesion's rapid onset, NSAID-responsive and reversible course, its poorly defined borders, and the eccentric periadventitial enhancement on MRI were inconsistent with stable atherosclerotic plaque. Whether this represents (i) idiopathic TIPIC arising coincidentally at a plaque site, or (ii) a plaque-associated/plaque-instability inflammatory process that mimics TIPIC, cannot be established from the present evidence; the distinction remains unresolved. Rather than categorically excluding plaque, we therefore discuss it as a possible local microenvironmental factor influencing the location of inflammation. (2) Subacute thyroiditis: Both conditions are self-limiting and can cause neck pain radiating to the ear/parotid region. However, subacute thyroiditis is often preceded by an upper respiratory infection (1–3 weeks prior). Thyroid ultrasound shows patchy hypoechoic areas, with elevated ESR/CRP, and thyroid function tests show elevated FT4/FT3 and with suppressed TSH (15). Treatment often involves antipyretics/NSAIDs (16). (3) Carotid artery dissection: It often has a history of mechanical injury/or trauma. It can also present with unilateral neck pain. Ultrasound typically shows a widened lumen, a flapping intimal flap creating true/and false lumens, possibly with intramural hematoma/thrombus, leading to stenosis/occlusion, and hemodynamic changes (17, 18). (4) Takayasu arteritis: It usually occurs in young females, is chronic, and typically requires steroids/immunosuppressants, with self-limitation being rare (19, 28). Ultrasound shows loss of normal wall structure, with diffuse, homogeneous, concentric wall thickening (“macaroni sign”) (20), luminal stenosis/occlusion, and possible thrombosis (21, 22).

Imaging is the gold standard for diagnosing TIPIC syndrome (23). Multiple studies have discussed the optimal technique for diagnosis and follow-up (1, 24, 25). CTA, MRI, and MRA all have unique features and advantages (23, 24, 26, 27). However, ultrasonography offers advantages of low cost, non-invasiveness, good repeatability, and high resolution in the diagnosis and follow-up of this disease. Current reports on this disease in the ultrasonography literature are relatively scarce. A multinational multicenter retrospective study of 72 patients confirmed the importance of ultrasound in follow-up (2). Rafailidis et al. (24) indicated that ultrasound is the preferred imaging method for evaluating TIPIC syndrome.

The clinical value of this case lies in documenting a reversible inflammatory lesion at an atypical, plaque-bearing carotid site, with bilateral symptoms arising from a unilateral lesion. Unlike the typical TIPIC phenotype — involvement of the distal carotid bifurcation and predominantly unilateral pain — this case occurred in the mid-portion of the common carotid artery in a patient with a pre-existing atherosclerotic plaque, and presented with bilateral neck pain despite a unilateral lesion. The case therefore underscores the need to include TIPIC in the differential diagnosis of neck pain even when a plaque is already known to be present at the symptomatic site, and to evaluate such patients with serial ultrasound.

This single-case report has several limitations. First, it is a case report rather than a controlled comparison, so the observations cannot establish prevalence or causal relationships. Second, MRI was performed only during the initial episode; because the patient declined further cross-sectional imaging, MRI was not repeated at recurrence, and the diagnosis of the second episode therefore rested on clinical and sonographic findings rather than on a repeated vessel-wall study. Third, no laboratory tests, including inflammatory markers, were repeated during the recurrence, leaving open whether the episode represented active inflammation or a non-inflammatory cause. Fourth, because residual wall thickening of approximately 2.5 mm was still present at the one-month follow-up, complete resolution before the second episode could not be demonstrated; the subsequent increase to approximately 3.1 mm may therefore represent true recurrence, persistent inflammation, or measurement variability. Finally, the proposed mechanism linking the pre-existing plaque to TIPIC via abnormally proliferated vasa vasorum is speculative and remains to be tested.

## Conclusion

This study did not seek to broadly expand the overall understanding of TIPIC syndrome. Rather, it refines and supplements the atypical lesional features associated with atherosclerotic plaque in this rare disease, and defines a clinical lesion pattern distinct from those reported in the existing literature. Unlike previous studies, which have predominantly focused on recurrent TIPIC at the carotid bifurcation, the present case exhibits two clearly differentiated lesional characteristics. First, the recurrence site is atypical: the patient experienced recurrence at the mid-portion of the left common carotid artery, rather than at the much more common carotid bifurcation. Second, the lesional pattern is specific: both episodes occurred superimposed on a pre-existing carotid atherosclerotic plaque.

Accordingly, this report adds a relatively uncommon phenotype — recurrent TIPIC at an atypical mid-common-carotid site, occurring in the setting of a pre-existing atherosclerotic plaque — to the limited TIPIC literature. It may serve as a detailed reference for future research on the vascular pathophysiology of this disease, for the clinical discrimination of TIPIC from plaque-related mimics, and for individualized management.

## Data Availability

The raw data supporting the conclusions of this article will be made available by the authors, without undue reservation.
